# On Flat Objects of Finitely Accessible Categories

**DOI:** 10.1155/2013/451091

**Published:** 2013-10-28

**Authors:** Septimiu Crivei

**Affiliations:** Faculty of Mathematics and Computer Science, “Babeş-Bolyai” University, Street Mihail Kogălniceanu 1, 400084 Cluj-Napoca, Romania

## Abstract

Flat objects of a finitely accessible additive category *𝒞* are described in terms of some objects of the associated functor category of *𝒞*, called strongly flat functors. We study closure properties of the class of strongly flat functors, and we use them to deduce the known result that every object of a finitely accessible abelian category has a flat cover.

## 1. Introduction

The famous Enochs's Flat Cover Conjecture played a key part in the development of the theory of module approximations, which has the root in the work of Auslander, Smalø, and Enochs [1, 2]. The conjecture stated that every module has a flat cover, and it was proved by Bican et al. [3, Theorem 3]. Afterwards, the problem was considered in various more general categories. For instance, Crivei et al. [4] and Rump [5] showed in two different ways that every object of a finitely accessible abelian category has a flat cover. Nevertheless, the knowledge about flat objects in such categories is rather limited. The present paper is intended to make a further step towards a better understanding of flat objects in finitely accessible additive categories.

It is well known that every finitely accessible additive category *𝒞* has an associated (Grothendieck) functor category (fp(*𝒞*)^op^, Ab) consisting of all contravariant additive functors from the full subcategory fp(*𝒞*) of finitely presented objects of *𝒞* to the category Ab of abelian groups. Moreover, Yoneda functor *H* : *𝒞* → (fp(*𝒞*)^op^, Ab), defined on objects by the assignment *X* ↦ *H*_*X*_ = Hom_*𝒞*_(−, *X*)|_fp(*𝒞*)_, induces equivalence between *𝒞* and the full subcategory of flat objects of (fp(*𝒞*)^op^, Ab). We are interested in determining the objects of the functor category (fp(*𝒞*)^op^, Ab) which correspond to flat objects in the original category *𝒞* via the above equivalence. These will be the so-called strongly flat objects of (fp(*𝒞*)^op^, Ab). We study some closure properties of the class of strongly flat objects, among which the closure under direct limits and pure epimorphic images. As an application, we use them to deduce the known result that every object of a finitely accessible abelian category has a flat cover. Note that every finitely accessible abelian category is already Grothendieck [6, Theorem 3.15].

## 2. Preliminaries

We recall some further terminology on finitely accessible additive categories, mainly following [6, 7]. Throughout the paper all categories and functors will be additive. An additive category *𝒞* is called *finitely accessible* if it has direct limits, the class fp(*𝒞*) of finitely presented objects is skeletally small, and every object is a direct limit of finitely presented objects. Let *𝒞* be a finitely accessible additive category. A *sequence* $0\to X\overset{f}{\to }Y\overset{g}{\to }Z\to 0$ in *𝒞* is a pair of composable morphisms with *gf* = 0. The above sequence in *𝒞* is called *pure exact* if it induces an exact sequence of abelian groups 0 → Hom_*𝒞*_(*P*, *X*) → Hom_*𝒞*_(*P*, *Y*) → Hom_*𝒞*_(*P*, *Z*) → 0 for every finitely presented object *P* of *𝒞*. This implies that *f* and *g* form a kernel-cokernel pair, in which *f* is called a *pure monomorphism* and *g* a *pure epimorphism*. The pure exact sequences in *𝒞* are those which become exact sequences in (fp(*𝒞*)^op^, Ab) through Yoneda embedding functor *H* : *𝒞* → (fp(*𝒞*)^op^, Ab), defined on objects by *X* ↦ *H*_*X*_ = Hom_*𝒞*_(−, *X*)|_fp(*𝒞*)_ and correspondingly on morphisms. The functor *H* preserves and reflects purity [6, Corollary 5.11] and commutes with direct limits. An object *Z* of *𝒞* is called *pure projective* if it is projective with respect to every pure exact sequence and *flat* if every epimorphism *Y* → *Z* is pure (e.g., see [6, 8]). If 0 → *A* → *B* → *C* → 0 is a pure exact sequence in (fp(*𝒞*)^op^, Ab) with *B* flat, then *A* and *C* are flat (e.g., see [6, Proposition 5.9] and [9, Proposition 36.1]).

By a *class* of objects in an additive category *𝒞* we mean a class of objects closed under isomorphisms. Let *M* be an object in *𝒞* and *𝒳* a class of objects in *𝒞*. Recall from [10] that a morphism *f* : *X* → *M* in *𝒞*, with *X* ∈ *𝒳*, is an *𝒳-precover* of *M* if the induced abelian group homomorphism Hom(*X*′, *f*) : Hom(*X*′, *X*) → Hom(*X*′, *M*) is an epimorphism for every *X*′ ∈ *𝒳*. An *𝒳*-precover *f* : *X* → *M* of *M* is an *𝒳-cover* if every endomorphism *g* : *X* → *X* with *fg* = *f* is an automorphism. The class *𝒳* is called *(pre)covering* if every object of *𝒞* has an *𝒳*-cover. Dually one defines the notions of relative *(pre)envelope* and *(pre)enveloping* class. For instance, every class of modules closed under direct products and pure submodules is preenveloping [11], whereas every class of modules closed under direct limits and pure epimorphic images is covering [4, 12].

## 3. Strongly Flat Objects in Functor Categories

We are interested in identifying certain objects of a finitely accessible additive category *𝒞* in terms of corresponding objects of its associated functor category through Yoneda functor *H* : *𝒞* → (fp(*𝒞*)^op^, Ab). To this end, we introduce and study a specialization of flatness in (fp(*𝒞*)^op^, Ab), which is different from a strongly flat functor in the sense of [13]. Recall that every flat object of (fp(*𝒞*)^op^, Ab) is of the form *H*_*Z*_ for some object *Z* of *𝒞*.

**Definition 1**

Let *𝒞* be a finitely accessible additive category. A flat object *H*_*Z*_ of (fp(*𝒞*)^op^, Ab) is called *strongly flat* if for every morphism *H*_*g*_ : *H*_*Y*_ → *H*_*Z*_ in (fp(*𝒞*)^op^, Ab) such that *g* : *Y* → *Z* is an epimorphism in *𝒞*, and for every finitely presented object *P* of (fp(*𝒞*)^op^, Ab), the induced abelian group homomorphism Hom(*P*, *H*_*g*_) : Hom(*P*, *H*_*Y*_) → Hom(*P*, *H*_*Z*_) is an epimorphism.

**Theorem 2**

Let *𝒞* be a finitely accessible abelian category. Then the class of strongly flat objects of ( fp (*𝒞*)^ op ^, Ab )  is closed under pure epimorphic images, extensions, direct sums, and direct limits.

**Proof**

Let 0 → *A* → *B* → *C* → 0 be a pure exact sequence in (fp(*𝒞*)^op^, Ab) with *B* strongly flat. Then *B* is flat, hence *A* and *C* are also flat. It follows that *A*≅*H*_*X*_, *B*≅*H*_*Y*_, and *C*≅*H*_*Z*_ for some objects *X*, *Y*, and *Z* of *𝒞*. Then the initial pure exact sequence has the form

(1) $$\begin{array}{c} 0\to H_{X}\overset{H_{f}}{\to }H_{Y}\overset{H_{g}}{\to }H_{Z}\to 0 \end{array}$$

for some morphisms *f*, *g* in *𝒞*. Now let *H*_*w*_ : *H*_*Z*′_ → *H*_*Z*_ be a morphism in (fp(*𝒞*)^op^, Ab) such that *w* : *Z*′ → *Z* is an epimorphism in *𝒞*, and let *P* be a finitely presented object of (fp(*𝒞*)^op^, Ab). Consider the pullback of *H*_*g*_ and *H*_*w*_ in (fp(*𝒞*)^op^, Ab) in order to obtain the following commutative diagram with exact rows:

 

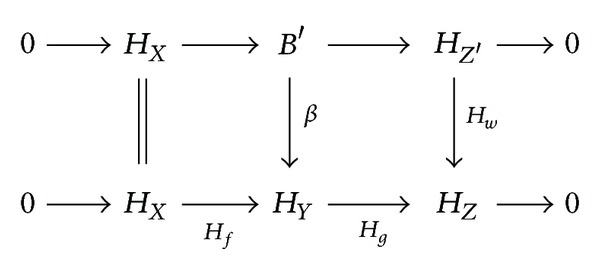
(2)
Since *H*_*X*_ and *H*_*Z*′_ are flat, so is *B*′. Hence *B*′≅*H*_*Y*′_ for some object *Y*′ of *𝒞*, and then *β* = *H*_*v*_ for some morphism *v* : *Y*′ → *Y* in *𝒞*. The full and faithful functor *H* reflects pullbacks [14, Chapter II, Theorem 7.1]. Since *𝒞* is abelian, pullbacks preserve epimorphisms; hence *v* is an epimorphism in *𝒞*. Since *H*_*Y*_ is strongly flat and is part of a pure exact sequence, Hom(*P*, *β*) and Hom(*P*, *H*_*g*_) are epimorphisms. Then the commutative diagram

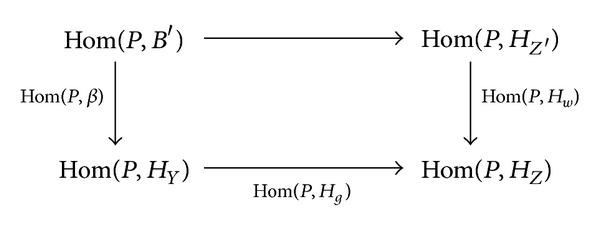
(3)
shows that Hom(*P*, *H*_*w*_) is an epimorphism. Hence *C*≅*H*_*Z*_ is strongly flat.

Now let 0 → *A* → *B* → *C* → 0 be a short exact sequence in (fp(*𝒞*)^op^, Ab) with *A* and *C* strongly flat. Then *A* and *C* are flat, and so *B* is also flat. It follows that *A*≅*H*_*X*_, *B*≅*H*_*Y*_, and *C*≅*H*_*Z*_ for some objects *X*, *Y*, and *Z* of *𝒞*. Then the initial short exact sequence has the form

(4) $$\begin{array}{c} 0\to H_{X}\overset{H_{f}}{\to }H_{Y}\overset{H_{g}}{\to }H_{Z}\to 0 \end{array}$$

for some morphisms *f*, *g* in *𝒞*, and it is pure by the flatness of *H*_*Z*_. Now let *H*_*v*_ : *H*_*Y*′_ → *H*_*Y*_ be a morphism in (fp(*𝒞*)^op^, Ab) such that *v* : *Y*′ → *Y* is an epimorphism in *𝒞*, and let *P* be a finitely presented object of (fp(*𝒞*)^op^, Ab). Consider the pullback of *H*_*f*_ and *H*_*v*_ in (fp(*𝒞*)^op^, Ab) in order to obtain the following commutative diagram with exact rows:

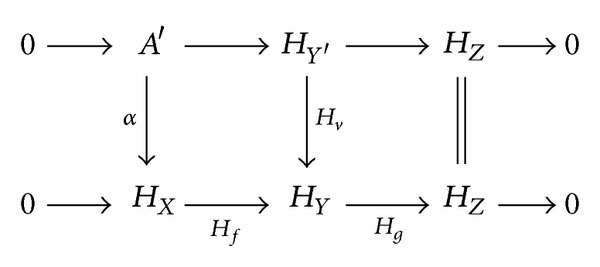
(5)
Since *H*_*Z*_ is flat, the upper row of the diagram is pure. Since *H*_*Y*′_ is flat, it follows that *A*′ is also flat. Hence *A*′≅*H*_*X*′_ for some object *X*′ of *𝒞*, and then *α* = *H*_*u*_ for some morphism *u* : *X*′ → *X* in *𝒞*. Using that *H* is full and faithful and *𝒞* is abelian, one deduces as in the first part of the proof that *u* is an epimorphism in *𝒞*. Since *H*_*X*_ is strongly flat, Hom(*P*, *α*) is an epimorphism. Then the induced commutative diagram with exact rows

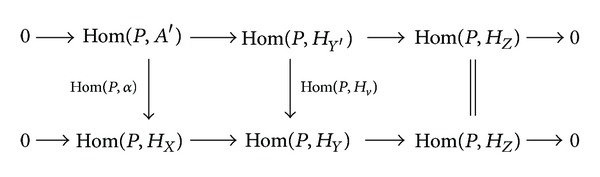
(6)
implies that Hom(*P*, *H*_*v*_) is an epimorphism. Hence *B*≅*H*_*Y*_ is strongly flat.

The closure of the class of strongly flat objects of (fp(*𝒞*)^op^, Ab) under extensions implies its closure under finite direct sums. Now let ⊕_*i*∈*I*_ *H*_*Z*_*i*__≅*H*_⊕_*i*∈*I*_*Z*_*i*__ be a direct sum of strongly flat objects of (fp(*𝒞*)^op^, Ab). Let *H*_*g*_ : *H*_*Y*_ → *H*_⊕_*i*∈*I*_*Z*_*i*__ be a morphism in (fp(*𝒞*)^op^, Ab) such that *g* : *Y* → ⊕_*i*∈*I*_ *Z*_*i*_ is an epimorphism in *𝒞*, and let *P* be a finitely presented object of (fp(*𝒞*)^op^, Ab). Then there is a finite subset *F* of *I* such that

(7) $$\begin{array}{c} \text{Hom}\left(P,\varphi \right):\text{Hom}\left(P,\underset{i\in F}{⨁}H_{Z_{i}}\right)\to \text{Hom}\left(P,\underset{i\in I}{⨁}H_{Z_{i}}\right) \end{array}$$

is an epimorphism, where *φ* : ⊕_*i*∈*F*_ *H*_*Z*_*i*__ → ⊕_*i*∈*I*_ *H*_*Z*_*i*__ is the inclusion morphism. Then *φ*≅*H*_*u*_ : *H*_⊕_*i*∈*F*_*Z*_*i*__ → *H*_⊕_*i*∈*I*_*Z*_*i*__, where *u* : ⊕_*i*∈*F*_ *Z*_*i*_ → ⊕_*i*∈*I*_ *Z*_*i*_ is the inclusion morphism. Consider the pullback of *u* and *g* in *𝒞*:

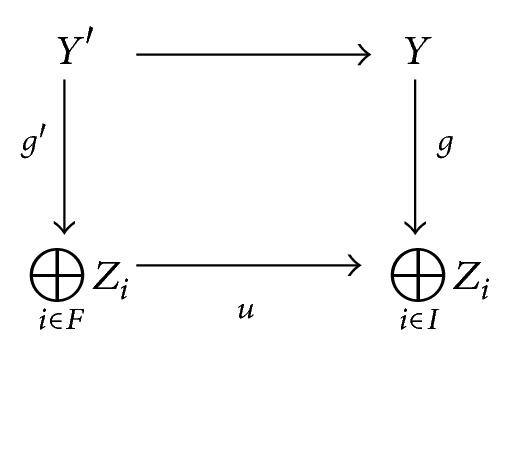
(8)
Since *𝒞* is abelian, *g*′ is an epimorphism in *𝒞*. Since ⊕_*i*∈*F*_ *H*_*Z*_*i*__ is strongly flat, it follows that Hom(*P*, *H*_*g*′_) : Hom(*P*, *H*_*Y*′_) → Hom(*P*, ⊕_*i*∈*F*_ *H*_*Z*_*i*__) is an epimorphism. Then the induced commutative diagram

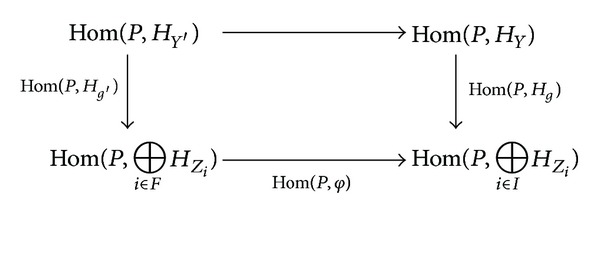
(9)
implies that Hom(*P*, *H*_*g*_) is an epimorphism. Hence ⊕_*i*∈*I*_ *H*_*Z*_*i*__ is strongly flat.

Finally, let (*H*_*Z*_*i*__, *f*_*ij*_)_*I*_ be a direct system of strongly flat objects of (fp(*𝒞*)^op^, Ab). Then there is a pure epimorphism

(10) $$\begin{array}{c} \underset{i\in I}{⨁}H_{Z_{i}}\to \underset{\to }{\lim}H_{Z_{i}} \end{array}$$

in (fp(*𝒞*)^op^, Ab) (e.g., see [9, Example 33.9]). We have already proved that the class of strongly flat objects of (fp(*𝒞*)^op^, Ab) is closed under direct sums and pure epimorphic images. Hence the direct limit $\underset{\to }{\lim}H_{Z_{i}}$ is strongly flat.

## 4. Flat Objects in Finitely Accessible Categories

Now let us relate flat objects of a finitely accessible additive category *𝒞* and strongly flat objects of its associated functor category (fp(*𝒞*)^op^, Ab).

**Theorem 3**

Let *𝒞* be a finitely accessible additive category. Then the equivalence induced by the Yoneda functor *H* : *𝒞* → ( fp (*𝒞*)^ op ^, Ab) between *𝒞* and the full subcategory of flat objects of ( fp (*𝒞*)^ op ^, Ab) restricts to equivalences between the following full subcategories:

1. pure-projective objects of *𝒞* and projective objects of ( fp (*𝒞*)^ op ^, Ab),
2. flat objects of *𝒞* and strongly flat objects of ( fp (*𝒞*)^ op ^, Ab),
3. projective objects of *𝒞* and strongly flat projective objects of ( fp (*𝒞*)^ op ^, Ab).

**Proof**

(1) By [7, Lemma 3.1].

(2) Assume first that *Z* is a flat object of *𝒞*. Let *H*_*g*_ : *H*_*Y*_ → *H*_Z_ be a morphism in (fp(*𝒞*)^op^, Ab) such that *g* : *Y* → *Z* is an epimorphism in *𝒞*, and let *γ* : *P* → *H*_*Z*_ be a morphism in (fp(*𝒞*)^op^, Ab) with *P* finitely presented. Since *Z* is flat in *𝒞*, *g* is a pure epimorphism, and so there is a pure exact sequence

(11) $$\begin{array}{c} 0\to X\to Y\overset{g}{\to }Z\to 0 \end{array}$$

in *𝒞*. Then the induced sequence

(12) $$\begin{array}{c} 0\to H_{X}\to H_{Y}\overset{H_{g}}{\to }H_{Z}\to 0 \end{array}$$

is pure exact in (fp(*𝒞*)^op^, Ab). Now *γ* lifts to a morphism *P* → *H*_*Y*_, showing that *H*_*Z*_ is strongly flat in (fp(*𝒞*)^op^, Ab).

Conversely, assume that *H*_*Z*_ is a strongly flat object of (fp(*𝒞*)^op^, Ab). Consider in *𝒞* an epimorphism *g* : *Y* → *Z*, a finitely presented object *L*, and a morphism *w* : *L* → *Z*. Then *H*_*L*_ is finitely generated projective and so finitely presented in (fp(*𝒞*)^op^, Ab) (e.g., see [15, Theorem 1.1]). Since *H*_*Z*_ is strongly flat in (fp(*𝒞*)^op^, Ab), there is a morphism *φ* : *H*_*L*_ → *H*_*Y*_ such that *H*_*g*_ *φ* = *H*_*w*_. Now we have *φ* = *H*_*h*_ for some morphism *h* : *L* → *Y* in *𝒞*. Then *gh* = *w*, showing that *g* : *Y* → *Z* is a pure epimorphism in *𝒞*, and so *Z* is flat in *𝒞*.

(3) This follows by (1) and (2).

Using the above theorems we may deduce the following known result on the existence of flat covers in finitely accessible abelian (Grothendieck) categories (see [4, Corollary 3.3] and [5, page 1604]).

**Corollary 4**

Let *𝒞* be a finitely accessible abelian category. Then the class of flat objects of *𝒞* is covering.

**Proof**

The class of strongly flat objects of the functor category (fp(*𝒞*)^op^, Ab) is closed under direct limits and pure epimorphic images by Theorem 2. Then it is a covering class in (fp(*𝒞*)^op^, Ab) by [4, Theorem 2.4] (also see [12, Theorem 2.5]). By Theorem 3 and [4, Lemma 2.5] it follows that the class of flat objects of *𝒞* is a covering class.
